# Genome-wide transcriptomics of the amygdala reveals similar oligodendrocyte-related responses to acute and chronic alcohol drinking in female mice

**DOI:** 10.1038/s41398-022-02231-2

**Published:** 2022-11-12

**Authors:** Sharvari Narendra, Claudia Klengel, Bilal Hamzeh, Drasti Patel, Joy Otten, Roy Lardenoije, Emily L. Newman, Klaus A. Miczek, Torsten Klengel, Kerry J. Ressler, Junghyup Suh

**Affiliations:** 1 Division of Depression and Anxiety Disorders, McLean Hospital, Department of Psychiatry, Harvard Medical School, Belmont, MA 02478 USA; 2 Department of Bioinformatics, Northeastern University, Boston, MA 02115 USA; 3 Department of Psychiatry and Psychotherapy, University Medical Center Göttingen, Göttingen, Germany; 4 Psychology and Neuroscience Departments, Tufts University, Medford, MA 02155 USA

**Keywords:** Molecular neuroscience, Addiction

## Abstract

Repeated excessive alcohol consumption is a risk factor for alcohol use disorder (AUD). Although AUD has been more common in men than women, women develop more severe behavioral and physical impairments. However, relatively few new therapeutics targeting development of AUD, particularly in women, have been validated. To gain a better understanding of molecular mechanisms underlying alcohol intake, we conducted a genome-wide RNA-sequencing analysis in female mice exposed to different modes (acute vs chronic) of ethanol drinking. We focused on transcriptional profiles in the amygdala including the central and basolateral subnuclei, brain areas previously implicated in alcohol drinking and seeking. Surprisingly, we found that both drinking modes triggered similar changes in gene expression and canonical pathways, including upregulation of ribosome-related/translational pathways and myelination pathways, and downregulation of chromatin binding and histone modification. In addition, analyses of hub genes and upstream regulatory pathways revealed that voluntary ethanol consumption affects epigenetic changes via histone deacetylation pathways, oligodendrocyte and myelin function, and the oligodendrocyte-related transcription factor, *Sox17*. Furthermore, a viral vector-assisted knockdown of Sox17 gene expression in the amygdala prevented a gradual increase in alcohol consumption during repeated accesses. Overall, these results suggest that the expression of oligodendrocyte-related genes in the amygdala is sensitive to voluntary alcohol drinking in female mice. These findings suggest potential molecular targets for future therapeutic approaches to prevent the development of AUD, due to repeated excessive alcohol consumption, particularly in women.

## Introduction

Alcohol use disorder (AUD) is a chronic relapsing brain disorder and a major public health concern in the United States, where the lifetime prevalence of AUD among adults is nearly 30% [1]. Notably, although AUD has been more common in men than women, women show a faster transition to dependence and suffer severe behavioral and physical impairments [2, 3]. Despite the disorder’s prevalence and severity, our understanding of the molecular and behavioral mechanisms that drive alcohol abuse, specifically from a gender perspective, is fragmented and there are few effective treatments for alcohol abuse. One of the hallmarks of AUD is a gradual increase in alcohol consumption over time [4]. This increase in alcohol intake is thought to result from neurobiological adaptation induced by repeated episodes of alcohol drinking [5]. Moreover, prolonged heavy alcohol exposure appears to cause progressive dysfunction in multiple brain areas, most notably changes in neuronal plasticity in the brain’s reward and stress systems, such as in the amygdala [6].

The amygdala is comprised of multiple interconnected nuclei nested deep in the temporal lobe in humans, and its structures and functions are well-conserved across species. It has been associated with both emotion and motivation, playing an essential role in processing aversive and appetitive valence [7–9]. Previous neuroimaging studies demonstrated that alcohol cues trigger amygdala activation which correlates with craving for alcohol in humans with AUD [10, 11]. In animal models, chronic alcohol exposure alters neuronal transmission in the central nucleus of the amygdala (CeA), and the neural activity of the CeA during alcohol withdrawal is associated with levels of alcohol drinking in alcohol-dependent rats [12, 13]. Furthermore, the activation of the basolateral amygdala (BLA) and its projections to the nucleus accumbens is necessary for cue-induced alcohol seeking behaviors [14].

As alcohol has broad systemic and molecular targets, identifying and characterizing transcriptional responses to alcohol in a brain region-specific manner is vital to our understanding of the molecular mechanisms underlying alcohol-related behaviors and AUD development and susceptibility [15–18]. Several studies have applied genomics to examine alcohol-induced transcriptional effects using chronic models of voluntary ethanol consumption and forced exposure through ethanol vapor in rodents [19–23]. The results from these studies included molecular targets, such as alterations in neuronal function and signal transduction, indicating that chronic ethanol exposure and withdrawal have prominent actions on gene expression in multiple brain areas including the prefrontal cortex. However, these prior studies using microarrays with pre-determined numbers of genes and forced alcohol exposure have not directly addressed the genome-wide transcriptional responses to repeated voluntary alcohol drinking that leads to the escalation of alcohol intake over time. Furthermore, these studies primarily examined male animals, resulting in an incomplete understanding of the molecular and behavioral mechanisms that drive higher alcohol intake in females. In addition, few studies investigated gene networks in the amygdala that are targeted by voluntary alcohol drinking, where molecular processes may underlie the development and maintenance of alcohol-drinking and seeking behaviors [24, 25].

To gain a better insight into gene expression alterations impacted by acute and chronic/repeated voluntary oral ethanol consumption, we subjected C57Bl/6J (B6) mice, an inbred strain that shows high alcohol consumption and preference. Specifically, we used B6 females that are known to self-administer higher amounts of alcohol than males under most conditions in order to parallel the findings from the current study with the existing literature with male mice but to differentially address alcohol-drinking driven transcriptional changes [26, 27]. We employed a 2-bottle choice ethanol drinking procedure, in which either a single bout or chronic intermittent access that has been shown to escalate ethanol intake over weeks in mice [27]. We then explored transcriptional changes in the amygdala that may underly a progressive increase in ethanol intake. We found that acute and chronic ethanol drinking induced similar network-level changes in gene expression, suggesting that a single episode of ethanol consumption substantially alters amygdala transcriptomes that may be long-lasting. Furthermore, we identified expression networks that correlated with the level of ethanol consumption and ethanol preference, suggesting mechanistic relationships between amygdala gene expression and behavioral readout. Our analyses also revealed that some of the most strongly correlated genes, including Sox17, are associated with myelination and oligodendrocyte differentiation. In addition, we used a viral vector-assisted knockdown of Sox17 gene expression in the amygdala and confirmed that Sox17 is involved in escalating alcohol intake over time. Together, our findings provide systems-level evidence of the relationships between voluntary alcohol drinking and oligodendrocyte-related gene networks within the amygdala.

## Materials and methods

For more detail, see *Supplemental Material: Supplementary Methods*.

### Animals

Two separate batches (*N* = 12 for the first and *N* = 18 for the second batches) of adult female C57BL/6J mice at 7 weeks of age were purchased from Jackson Laboratories (Bar Harbor, ME) and kept under standard conditions with 12:12 h light/dark cycle (lights on: 07:00). Animals were group housed upon arrival and acclimated for 1–2 weeks. Then, mice were individually housed and allowed access to tap water and free (*ad libitum*) access to standard laboratory chow during the entire experimental period. All experiments were approved by and carried out in accordance with the Institutional Animal Care and Use Committee at McLean Hospital. All experimental and animal care procedures met the guidelines outlined in the NIH Guide for the Care and Use of Laboratory Animals. All efforts were made to minimize distress and the number of animals used.

### Alcohol drinking procedures

**Fig. 1:**
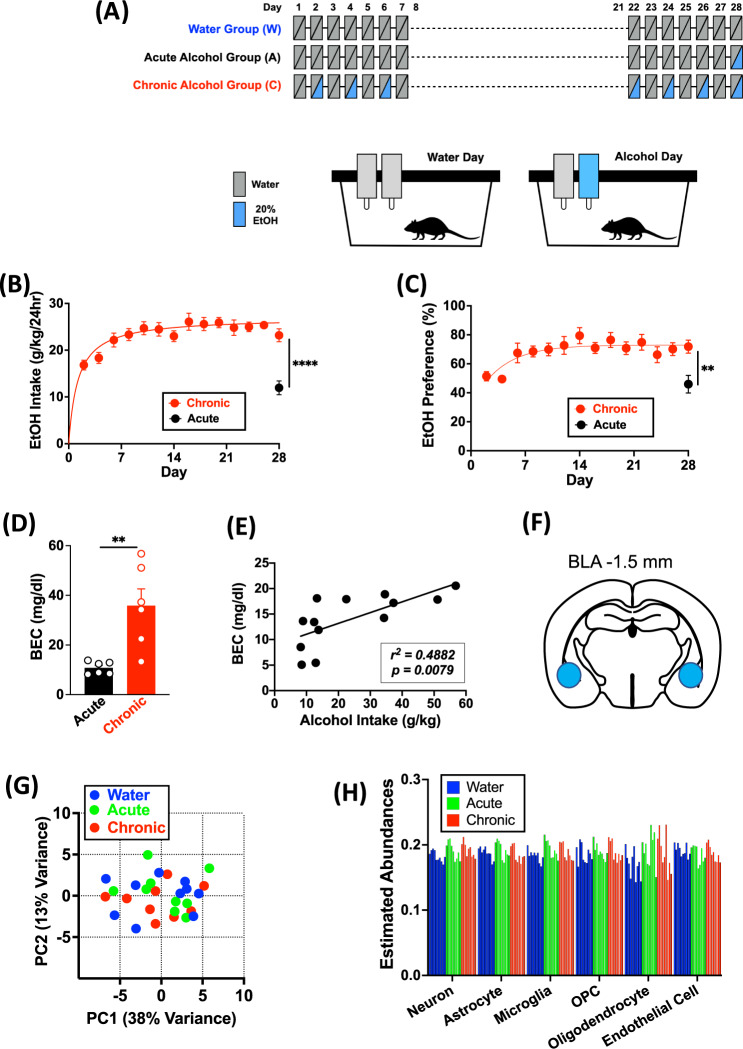
Experimental design, fluid consumption levels, and initial assessment of sequencing results. **A** Experimental design and timeline. **B** Ethanol intake over 24 h on water/20% EtOH drinking days. **C** EtOH preference ratios. **D** BEC measured in Acute and Chronic Drinking groups of 2nd cohort of mice on Day 28. **E** Correlation of BEC to the amount of ethanol consumed by mice in Acute and Chronic Drinking groups on Day 28. Data are mean ± SEM. **p < 0.01 and ****p < 0.0001 difference between groups. **F** Diagram for amygdala tissue collection. **G** PCA plot showing no separation on alcohol drinking condition over the first two principal components. **H** Bar graphs showing the estimated cell type abundance for seven relevant cell types as determined by cell type deconvolution analysis. Each bar represents a single sample.

Twenty percent of ethanol solution (v/v) was prepared in tap water from 95% ethyl alcohol (Pharmaco-AAPER, Brookfield, CT). Mice were changed to individual housing at least 24 h before the presentation of two 50-ml plastic centrifuge tubes of water on the metal wire cage lid for 2 days for acclimation to drinking from no. 6 rubber stoppers containing stainless steel ball-bearing sippers (Ancare, Bellmore, NY). Centrifuge tubes were securely held through the metal wire cage lid and presented to mice 2 h before the dark cycle and weighed to the nearest hundredth of a gram, 24 h after the fluids were given, and the left/right position of the tubes were alternated before each ethanol drinking session to avoid side preferences. To control for spillage and evaporation, daily “drip” averages (loss of fluid in two cages with no animal present) were subtracted from individual fluid intakes. Mice were also weighed weekly to the nearest tenth of a gram to calculate the grams of ethanol intake per kilogram of body weight. Preference for ethanol was calculated for ethanol solution compared with water, with formula being volume of ethanol intake (ml) divided by total volume fluid intake (ml). Mice from each cohort were assigned to three drinking groups. Mice in the acute drinking group (Acute Drinking) were given two centrifuge tubes of water for 27 days, then a tube with 20% ethanol and a tube with water on Day 28. The chronic intermittent access drinking group (Chronic Drinking) of mice received free-choice 24 h access to 20% ethanol and water on every-other-day (EOD) basis for 4 weeks (28 days). Mice in the water drinking group (Water Drinking) received the same schedule of total fluid access but consumed only water from two tubes (Fig. 1A).

### Tissue collection

After completion of the experiments on day 29, 2 h into the light cycle, mice were sacrificed by decapitation following deep anesthetization with isoflurane. In order to minimize gene expression alterations induced by alcohol withdrawal and isoflurane exposure, mice in Acute and Chronic groups were kept in their home cages with two tubes (alcohol and water), and then transferred to an isoflurane chamber where they were anesthetized for less than 2 min. Trunk blood was collected in EDTA tubes to measure blood ethanol concentration (BEC), and vaginal smear was collected on non-coated glass microscope slides to determine the stage of the estrous cycle. Brains were rapidly removed from the skull, and placed on dry ice, and stored at −80 °C until further processing. Fresh frozen brains were sectioned at a thickness of 300 μm, then micropunches (1 mm in diameter and 1 mm in thickness) were aimed to include the following coordinates: ML ±3.2, AP −1.5, DV −5.0 mm, and bilaterally collected from the entire amygdala including basolateral amygdala (BLA) and central amygdala (CeA) based on established anatomical coordinates from the mouse brain atlas [28]. All the samples were placed in microcentrifuge tubes (1.5 ml), kept frozen in dry ice, and stored at −80 °C until RNA isolation. BEC was determined using the Analox Analyzer (Analox Instruments Inc., Lunenburg, MA) from blood samples (30 μl). Due to the lack of clear plasma separation after centrifugation, BEC measurement from the first cohort was not carried out. The vaginal cytology was carried out using crystal violet staining [29].

### RNA extraction and sequencing

Total RNA was isolated and purified using the Absolutely RNA Miniprep Kit (Agilent Technologies, Santa Clara, CA) according to the manufacturer’s protocol. The quality and concentration of the extracted RNA were evaluated using a NanoDrop 8000 spectrophotometer (ThermoFisher Scientific, Waltham, MA). As one mouse from each cohort was removed due to a technical error, 11 samples from the first cohort (4 from Water, 4 from Acute and 3 from Chronic Drinking groups) and 17 samples from the second cohort (6 from Water, 5 from Acute and 6 from Chronic Drinking groups) were sent to BGI (Hong Kong, China) for sequencing. Library construction and whole genome sequencing were conducted on the BGISEQ-500 platform using the DNBseq short-read 100 bp paired-end reads with the sequencing depth of 50 million. On average, 46–52 million raw reads per sample were achieved.

### Bioinformatics analyses

We performed multiple bioinformatic analyses including differential expression analysis, pathway enrichment analysis, GWAS Catalog, and DisGeNET comparison, and identification of hub genes and regulatory transcription factors. Detailed analyses are provided in Supplementary Methods.

### Quantitative PCR (qPCR)

RNA samples were reverse transcribed into cDNA using superscript IV kit (ThermoFisher Scientific, Waltham, MA) using random hexamer primers. Complementary DNA was amplified on a ViiA7 Real-Time PCR system (ThermoFisher Scientific, Waltham, MA) with POWRUP SYBR Green Master Mix (ThermoFisher Scientific, Waltham, MA). Primers for genes of interest and the housekeeping gene *Actb* are described in Supplementary Table 1. Specificity of the qPCR reaction was confirmed with melt curve analysis to ensure that only the expected PCR product was amplified. Duplicates were run for each reaction, and Ct values were normalized using the established delta-delta Ct method (2−∆∆Ct) and then normalized to *Actb* Cts.

### Viral-mediated gene transfer

Lentivirus encoding Sox17-shRNA was purchased (Santa Cruz Biotechnology) and bilaterally microinjected into the amygdala of B6 female mice that were subsequently subjected to the EOD drinking paradigm. Detailed surgical procedures are provided in Supplementary Methods.

### Statistical analysis

Data were analyzed using R (version. 4.0.0) or GraphPad Prism (version 9.1, GraphPad Software, San Diego, CA). The level of significance was set at *p* < 0.05, and results are presented as mean plus or minus standard error of the mean (*M* ± SEM). For the drinking data, ethanol intake (g/kg), volume (ml) of water and ethanol consumed, total fluid intake (ml), ethanol preference (%), and body weight (g) were analyzed with multiple two-way analyses of variance (ANOVAs), followed by Bonferroni post hoc analysis when significant group effects were found (*p* < 0.05). BEC (mg/dl) and single daily ethanol intake (g/kg) on day 29 between Acute and Chronic Drinking groups were analyzed with one-way ANOVA. Differences in cell type proportions were assessed with 2 sample t-test.

## Results

### 2-bottle choice EOD drinking

We employed a well-established 2-bottle choice every-other-day (EOD) drinking paradigm [27] and divided two independent batches of mice into 3 drinking groups (Water, Acute, and Chronic) (Fig. 1A). Ethanol intake of the first batch was slightly higher than that of the second batch in both Acute and Chronic Drinking groups but the small difference was not statistically significant (*p* = 0.7313, Supplementary Fig. 1A). Consistent with previous behavioral studies [27, 30], mice in the Chronic Drinking group increased ethanol intake across the first 2-week period, subsequently maintaining a stable level (a daily average of 25.14 ± 0.50 g/kg in weeks 3–4). On day 28, mean ethanol intake was 23.19 ± 1.62 g/kg for the Chronic Drinking group, which was significantly higher (*p* < 0.0001) than that of the Acute Drinking group (11.96 ± 2.36 g/kg) (Fig. 1B). As expected, there were no differences in a daily average of total liquid consumption across 4 weeks between the groups (Water, 4.80 ± 0.41 ml; Acute, 4.57 ± 0.41 ml; Chronic, 4.45 ± 0.38 ml) (Supplementary Fig. 1B). Consequently, 24 h preference values revealed that mice in the Chronic Drinking group showed increased preference as early as after 1 week of intermittent drinking, which was sustained at an average 72.60% in weeks 3–4. In contrast, mice in the Acute Drinking group displayed 45.98% preference on the first and only day of alcohol access, Day 28 (Fig. 1C). During 4 weeks of the drinking period, body weight (g) did not show any significant group differences on day 1 (Water, 19.60 ± 0.51; Acute, 19.63 ± 0.36; Chronic, 19.71 ± 0.32) and day 29 (Water, 20.83 ± 0.47; Acute, 20.54 ± 0.23; Chronic, 20.81 ± 0.41). On the final day of the study, BECs of Chronic Drinking group were significantly higher than those of Acute Drinking group, as BECs positively correlated with ethanol intake levels (Fig. 1D, E). In addition, all the mice in each drinking group were in either proestrus or estrus phase of the estrous cycle (6 proestrus and 4 estrus in Water; 6 proestrus and 3 estrus in Acute; 6 proestrus and 3 estrus in Chronic Drinking group). There was no difference in alcohol consumption on Day 28 among mice in different phases of the estrous cycle in Acute and Chronic Drinking groups (Acute, proestrus vs estrus, *p* = 0.9574; Chronic, proestrus vs estrus, *p* = 0.9964), which is consistent with previous findings that alcohol intake is not affected by estrous cycle phase in female rodents [31, 32]. These data demonstrate that our protocol succeeded in achieving standard levels of both acute and chronic drinking behaviors for subsequent transcriptional profiling of amygdala function.

### RNA-seq analysis

Since both acute and chronic alcohol consumption can lead to gene expression alterations and cellular adaptations, RNA sequencing (RNA-seq) analysis was used to determine genome-wide transcriptomic profiles. Given the important roles of the CeA and BLA in alcohol-related synaptic changes and behaviors, we collected micropunches containing these subnuclei from the three drinking groups (Fig. 1F). We detected *n* = 49,477 transcripts in our experiment. Filtering of low expression genes (<10 reads) results in the exclusion of *n* = 33,217 transcripts. Since we collected RNA samples from two independent batches of mice, we first used principal component analysis (PCA) of remaining *n* = 16,260 genes as input to determine the overall structure of the expression dataset between batches. The results revealed 89% of total variance and segregated clustering by batch (Supplementary Fig. 2A). We next investigated whether transcriptional effects were consistent between these two batches and found that expression changes for the top 100 genes from Acute Drinking group in batch 1 showed significant positive correlation with the same genes from Acute Drinking group in batch 2. Conversely, we also observed significant positive correlation when comparing changes for the top 100 genes in batch 2 with the corresponding genes in batch 1 (Supplementary Fig. 2B). Similarly, significant positive correlation was also seen between batch 1 and batch 2 in Chronic Drinking group (Supplementary Fig. 2C). The results indicate that an overall alcohol drinking-induced effect on these genes is coherent in both batches.

To adjust for the batch effects and to increase statistical power, we next used Combat-seq in R [33], a recent extension of the original ComBat adjustment framework [34] (Supplementary Fig. 2A). We confirmed no outliers isolated by experimental conditions along first two principal components with 51% of the total variance (Fig. 1G and Supplementary 2D). Furthermore, since differences in cell type proportions can be a major source of variation in gene expression profiles, we used a computational cell type deconvolution tool, BRETIGEA [35] to estimate the abundances of six relevant cell types, including neurons, astrocytes, microglia, oligodendrocyte precursor cells (OPC), oligodendrocytes, and endothelial cells. There were no significant differences among Drinking groups (*p* = 0.1006, Water vs Acute; *p* = 0.1528, Water vs Chronic; *p* = 0.2116, Acute vs Chronic) (Fig. 1H). Moreover, to confirm no differences in cell type proportions, we also used a different computational deconvolution tool, CIBERSORT, based on mouse cell type-specific markers from a previous study [36] (*p* > 0.9999, Water vs Acute; *p* > 0.9999, Water vs Chronic; *p* > 0.9999, Acute vs Chronic) (Supplementary Fig. 3A). Notably, both digital deconvolution methods revealed similar or larger contributions of oligodendrocytes and OPC to cell type abundance, which, we speculated, may be due to reference data sets that we used to run the methods. Nonetheless, these findings suggest that most of the observed variation in gene expression can be attributed to alcohol consumption rather than other confounding factors.

To identify genes exhibiting significantly altered expression due to alcohol drinking, we next calculated the expression level of each transcript based on the number of transcripts per million reads. Using the DESeq2 package with default parameters, we identified 1300 and 1384 differentially expressed genes between Acute and Water Drinking groups and between Chronic and Water Drinking groups, respectively. Then, we used the more stringent false discovery rate (FDR)-adjusted *p*-value cutoff of 0.05 to trim potential false positive results. We further identified 29 (Acute vs. Water, upregulated: 23 and downregulated: 6) and 97 (Chronic vs. Water, upregulated: 36 and downregulated: 61) differentially expressed genes (Table 1 and Supplementary Fig. 3B). These results provide evidence for robustly and significantly differentially expressed genes in the amygdala as a result of acute or chronic ethanol voluntary drinking.

**Table 1:** Top 30 differentially expressed genes (DEGs) in the Acute and Chronic Drinking groups.

Drinking condition	Gene symbol	Gene name	Fold change (log2)	*p* val	adj. *p* val
Acute	*4933427D14Rik*	Hypothetical protein LOC9851	−0.487	5.08E−08	0.001
	*Haghl*	Hydroxyacylglutathione Hydrolase-Like Protein	0.178	1.81E−07	0.001
	*Klf2*	Kruppel Like Factor 2	0.330	5.52E−06	0.016
	*Kat6a*	Lysine Acetyltransferase 6A	−0.152	6.87E−06	0.016
	*Btbd6*	BTB Domain Containing Protein 6	0.150	7.82E−06	0.016
	*Lrrc24*	Leucine Rich Repeat Containing Protein 24	0.188	8.48E−06	0.016
	*Mt1*	Matrix Metallopeptidase 14	0.208	8.76E−06	0.016
	*Kdm3a*	Lysine Demethylase 3A	−0.167	1.45E−05	0.022
	*Faap20*	Fanconi Anemia Core Complex Associated Protein 20	0.217	1.85E−05	0.022
	*Celf1*	CUGBP Elav-Like Family Member 1	−0.117	1.86E−05	0.022
	*Zwint*	ZW10 Interacting Kinetochore Protein	0.108	1.88E−05	0.022
	*Nts*	Neurotensin	0.548	2.20E−05	0.023
	*Kansl1*	KAT8 Regulatory NSL Complex Subunit 1	−0.104	2.96E−05	0.029
	*Cltb*	Clathrin Light Chain B	0.131	3.88E−05	0.035
	*Dusp26*	Dual Specificity Phosphatase 26	0.152	4.32E−05	0.036
	*Nfkbia*	NF-Kappa-B Inhibitor Alpha	0.253	4.45E−05	0.036
	*Srxn1*	Sulfiredoxin 1	0.141	5.31E−05	0.039
	*Btg2*	GF-Inducible Anti-Proliferative Protein PC3	0.425	5.42E−05	0.039
	*Rpl29*	Ribosomal Protein L29	0.138	6.26E−05	0.041
	*Diras1*	DIRAS Family GTPase 1	0.136	6.38E−05	0.041
	*Chga*	Chromogranin A	0.162	8.70E−05	0.046
	*Flywch2*	FLYWCH Family Member 2	0.411	8.95E−05	0.046
	*Pcsk1n*	Proprotein Convertase Subtilisin/Kexin Type 1 Inhibitor	0.144	8.98E−05	0.046
	*Lars2*	Leucyl-TRNA Synthetase 2, Mitochondrial	0.283	9.00E−05	0.046
	*Ndufa10*	NADH:Ubiquinone Oxidoreductase Subunit A1	0.098	1.03E−04	0.049
	*Vstm5*	V-Set And Transmembrane Domain-Containing Protein 5	0.241	1.06E−04	0.049
	*Ppp1r3f*	Protein Phosphatase 1 Regulatory Subunit 3F	0.169	1.07E−04	0.049
	*Slc39a11*	Solute Carrier Family 39 Member 11	0.183	1.12E−04	0.049
	*Xkr4*	XK Related 4	−0.380	1.15E−04	0.049
	*Dok5*	Docking Protein 5	0.189	1.25E−04	0.052
Chronic	*Kdm3a*	Lysine Demethylase 3A	−0.235	1.24E−09	0.000
	*Haghl*	Hydroxyacylglutathione Hydrolase-Like Protein	0.206	1.33E−09	0.000
	*Diras1*	DIRAS Family GTPase 1	0.172	3.92E−07	0.001
	*Lrrc24*	Leucine Rich Repeat Containing Protein 24	0.213	4.05E−07	0.001
	*Pcsk1n*	Proprotein Convertase Subtilisin/Kexin Type 1 Inhibitor	0.176	1.50E−06	0.003
	*Itpk1*	Inositol-Tetrakisphosphate 1-Kinase	0.205	4.71E−06	0.007
	*Cltb*	Clathrin Light Chain B	0.141	8.83E−06	0.009
	*Eml2*	Echinoderm MT-Associated Protein (EMAP)-Like Protein 2	0.191	9.57E−06	0.009
	*Kat6a*	Lysine Acetyltransferase 6A	−0.149	1.00E−05	0.009
	*Doc2a*	Double C2 Domain Alph	0.176	1.11E−05	0.009
	*Arhgap29*	Rho GTPase Activating Protein 29	−0.228	1.16E−05	0.009
	*Larp4*	La Ribonucleoprotein 4	−0.147	1.72E−05	0.012
	*Ubxn2b*	UBX Domain Protein 2B	−0.168	1.82E−05	0.012
	*Inafm1*	InaF Motif Containing 1	0.200	2.00E−05	0.012
	*Sp4*	Sp4 Transcription Factor	−0.172	3.49E−05	0.019
	*Mt1*	Matrix Metallopeptidase 14	0.194	3.56E−05	0.019
	*Arhgap20*	Rho GTPase Activating Protein 20	−0.210	3.92E−05	0.019
	*Ankrd13d*	Ankyrin Repeat Domain 13D	0.126	4.18E−05	0.019
	*Dgkb*	Diacylglycerol Kinase Beta	−0.159	4.37E−05	0.019
	*Enho*	Energy Homeostasis-Associated Protei	0.199	4.75E−05	0.020
	*Usp53*	Ubiquitin Specific Peptidase 53	−0.197	5.10E−05	0.020
	*Osbpl8*	Oxysterol Binding Protein Like 8	−0.210	5.58E−05	0.021
	*Btbd6*	BTB/POZ Domain-Containing Protein 6	0.135	5.93E−05	0.022
	*Nenf*	Neudesin Neurotrophic Factor	0.241	6.77E−05	0.024
	*Cep295*	Centrosomal Protein 295	−0.186	7.22E−05	0.024
	*Tmem106b*	Transmembrane Protein 106B	−0.119	7.31E−05	0.024
	*Hipk3*	Homeodomain Interacting Protein Kinase 3	−0.114	7.58E−05	0.024
	*Pcdhgb7*	Protocadherin Gamma Subfamily B, 7	−0.248	7.95E−05	0.024
	*Fnip1*	Folliculin Interacting Protein 1	−0.128	9.00E−05	0.025
	*Med1*	Mediator Complex Subunit 1	−0.132	9.17E−05	0.025

DEGs were identified using DESeq2 with default parameters including a nominal *p*-value cutoff of 0.05 (*p*-val). Then, to trim potential false positive results, FDR-adjusted *p*-value (adj. *p*-val) was used. Top 30 DEGs were ranked by adj. *p*-val.

### Validation of DEGs by qPCR

To validate the expression profiles obtained by bulkRNA-seq, seven genes including brain cytoplasmic RNA 1 (*Bc1*), BTG anti-proliferation factor 2 (*Btg2*), hydroxyacylglutathione hydrolase like (*Haghl*), leucine rich repeat containing 24 (*Lrrc24*), neudesin neurotrophic factor (*Nenf*), and lysine demethylase 3A (*Kdm3a*) were used for qPCR (Supplementary Tables 1 and 2). We selected these genes because (1) they displayed |Log2(fold change)| >0.2 in the Chronic Drinking group, (2) their transcripts are detected in mouse brain (Allen Brain Institute), and 3) some of genes including *Bc1*, *Btg2*, *Nenf*, and *Nts* have previously been shown to play roles in synaptic plasticity [37], neuronal proliferation [38], anxiety [39], and alcohol drinking [40], respectively. Consistent with the RNA-seq findings, in all cases, the relative fold change of gene expression was in the same direction in Acute and Chronic Drinking groups.

### Probing cell type-specificity signature of DEGs

BulkRNA-seq yields an averaged gene expression signals across all different cell types. In recent years, more evidence on the cellular heterogeneity in response to alcohol exposure is emerging. Although our in-silico approach provides evidence for a stable relative ratio of the different cell types among the drinking groups (Fig. 1H and Supplementary Fig. 3A), we next addressed cellular heterogeneity responding to alcohol exposure. By leveraging previous bulk and single cell RNA sequencing data sets collected from mouse and human tissues as reference single-cell type-specific expression profiles [36, 41–43], we asked if any of DEGs in our studies was included in the list. We found that some of DEGs were enriched in genes with glia annotation, particularly astrocyte and oligodendrocyte annotation (Supplementary Table 3), suggesting that alcohol drinking alters glia-related gene expression in the amygdala.

### GO and KEGG gene enrichment analyses

**Fig. 2:**
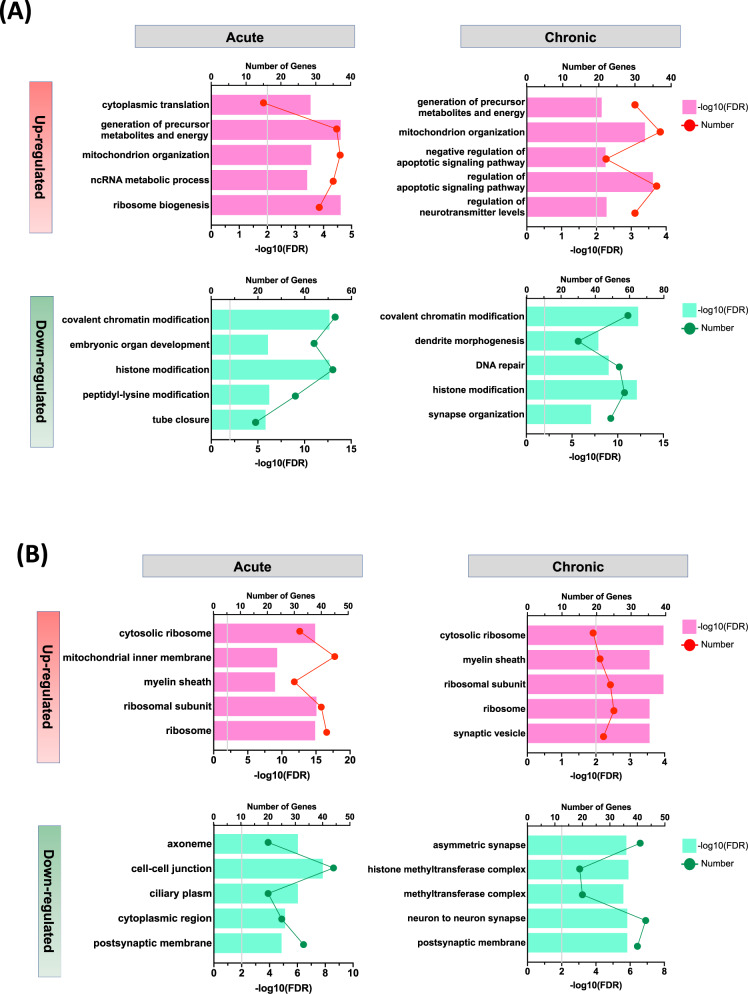
GO enrichment analysis of DEGs in response to Acute and Chronic alcohol drinking. The ordinate represents the GO terms, the upper abscissa indicates the number of genes in the GO terms, and the lower abscissa indicates the level of significance of the enrichment (gray bar, FDR = 0.01). **A** Genes were categorized with the Biological Process domain. **B** Genes were categorized with the Cellular Component domain.

To further identify networks of coordinately regulated genes that might point to alcohol-related specific biological functions, we next performed GO and KEGG Pathway enrichment analyses using the lists of DEGs with the criteria of *p*-value < 0.05, which was set to increase the number of genes in each drinking condition. We found that the primary effects of acute and chronic alcohol drinking were related to ribosome, cytoplasmic translation, chromatin binding, histone modification pathways, and various neurological diseases (Fig. 2A, B and Supplementary Fig. 4A, B). Interestingly, among those GO enrichment terms, “myelin sheath” (FDR-adjusted *p* = 0.0003) was in the top 5 upregulated pathways, suggesting alcohol drinking affects molecular and cellular mechanisms underlying oligodendrocyte maturation and myelination, consistent with our results from cell type-specificity comparison and previous reports that demonstrated glial dysfunction in AUD pathophysiology [44]. These findings suggest that alcohol drinking, particularly in repeated access, induces neuroadaptations mediated by glia-specific molecular alterations in the amygdala.

### Identification of the potential regulatory pathways

To identify a list of hub genes, we next used STRING using identified DEGs (FDR *p* < 0.05) as inputs, a common online database for predicting protein-protein interaction networks [45]. It revealed hub genes with 4–5 nodes, including histone deacetylase 2 (*Hdac2*), heterogeneous nuclear ribonucleoprotein M (*Hnrnpm*), histone deacetylase complex subunit sin3a (*Sin3a*), and chromodomain helicase DNA binding protein 1 (*Chd1*), particularly in the Chronic Drinking condition (Supplementary Fig. 5). The *Hdac2, Sin3a*, and *Chd1* are members of proteins associated with histone deacetylase activity. Notably, the expression of these genes in both Acute and Chronic Drinking groups was decreased, although not statistically significantly. qPCR also confirmed the relative changes in the same direction (Supplementary Tables 1 and 2). To determine upstream regulators of identified DEGs in current study, we next applied DEGs to the GeneGo MetaCore online database and identified 4 and 17 candidate upstream regulatory transcription factors in Acute and Chronic Drinking groups, respectively (Table 2). SRY-box transcription factor 17 (*Sox17*) stood out in both Acute and Chronic Drinking groups. Since *Sox17* has been shown to regulate oligodendrocyte progenitor cell expansion and differentiation, this finding is consistent with our results from GO/KEGG pathway analyses and cell type-specificity comparison (Fig. 2, Supplementary Fig. 4, and Supplementary Table 3). Together, these findings suggest that voluntary alcohol consumption affects epigenetic changes via histone deacetylation pathways, and oligodendrocyte-related transcriptional factor, *Sox17*.

**Table 2:** Candidate upstream transcription factors based on DEGs associated with the Acute and Chronic Drinking groups.

	Drinking groups				
Drinking condition	Gene symbol	Gene name	Actual targets	*p*-val	z-score
Acute	*Taf3*	TATA-Box Binding Protein Associated Factor 3	2	1.04E−04	16.02
	*c-Jun*	Jun Proto-Oncogene, AP-1 Transcription Factor Subunit	7	2.94E−04	5.76
	*c-Fos*	Fos Proto-Oncogene, AP-1 Transcription Factor Subunit	5	1.87E−04	5.27
	*Sox17*	SRY (Sex Determining Region Y)-Box Transcription Factor 17	22	3.60E−04	3.63
Chronic	*Rbpj*	Recombination Signal Binding Protein for Immunoglobulin Kappa J Region	58	2.27E−11	7.18
	*Sox17*	SRY (Sex Determining Region Y)-Box Transcription Factor 17	69	4.86E−11	6.70
	*Foxp3*	Forkhead Box P3	57	4.62E−08	5.69
	*Tal1*	TAL BHLH Transcription Factor 1, Erythroid Differentiation Factor	61	9.83E−08	5.45
	*Creb1*	CAMP Responsive Element Binding Protein 1	44	1.29E−07	5.74
	*Ets1*	ETS Proto-Oncogene 1, Transcription Factor	53	9.50E−07	5.09
	*c-Myc*	MYC Proto-Oncogene, BHLH Transcription Factor	37	6.78E−05	4.22
	*Runx1*	Runt-Related Transcription Factor 1	46	1.17E−04	3.94
	*Gata-2*	GATA Binding Protein 2	23	1.63E−04	4.20
	*E2f1*	E2F Transcription Factor 1	37	1.79E−04	3.92
	*Zfx*	Zinc Finger Protein X-Linked	23	2.20E−04	4.10
	*Gabp*	GA Binding Protein Transcription Factor Subunit Alpha	35	3.97E−04	3.69
	*Cebpe*	CCAAT/Enhancer Binding Protein (C/EBP), Epsilon	4	6.07E−04	5.85
	*Yy1*	YY1 Transcription Factor	13	7.79E−04	3.95
	*Ash2*	ASH2 Like, Histone Lysine Methyltransferase Complex Subunit	21	2.54E−03	3.21
	*Glis3*	GLIS Family Zinc Finger 3	20	2.82E−03	3.19
	*Klf9*	Kruppel Like Factor 9	3	3.47E−03	4.93

DEGs were used with GeneGo MetaCore to detect upstream transcription factors, which were ranked by z-score. 4 and 17 upstream transcription factors were identified in the Acute and Chronic Drinking groups, respectively.

### Validation of Sox17 regulation of alcohol drinking

**Fig. 3:**
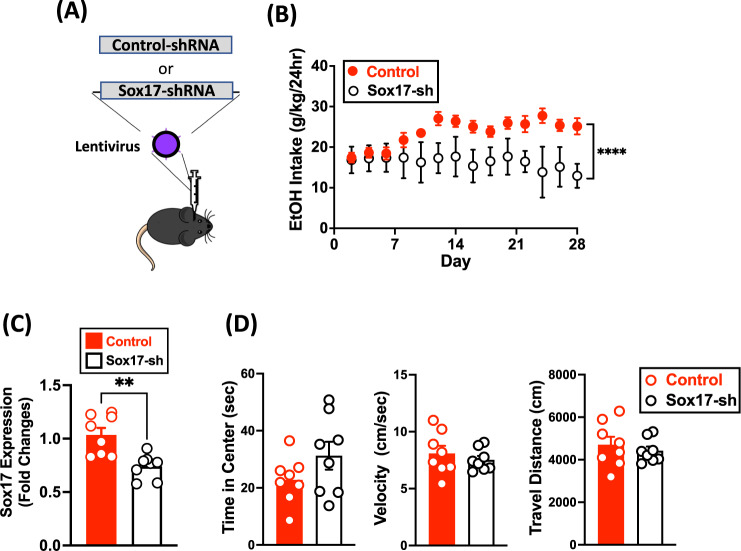
Downregulation of Sox17 in the amygdala reduces alcohol consumption. **A** Schematic illustrating the surgical strategy with a lentivirus encoding Sox17-shRNA. **B** Alcohol intake of mice bilaterally injected with either control or Sox17-shRNA lentivirus into the amygdala. The injection was conducted 2 weeks before subjecting the animals to EOD paradigm. **C** Changes of Sox17 expression were examined by qPCR in Sox17 shRNA- or control lentivector-injected mice. **D** Downregulation of Sox17 expression does not lead to significant changes in anxiety (time spent in the center of an open field) or locomotion (speed and travel distance). Data are mean ± SEM. **p < 0.01 and ****p < 0.0001 difference between groups.

To validate and further determine the role of Sox17 in escalated alcohol drinking, we microinjected lentiviral vectors encoding Sox17 shRNA into the amygdala of B6 female mice, thereby silencing the expression of Sox17 within this region (Fig. 3A). Two weeks after injections, animals were subjected to the EOD drinking paradigm. We found that the level of alcohol intake in Sox17 shRNA-injected animals (a daily average of 16.28 ± 1.47 g/kg) were significantly lower than that of control vector-injected mice (a daily average of 23.70 ± 3.36 g/kg), indicating that downregulation of Sox17 in the amygdala resulted in reduction of alcohol drinking (*P* < 0.0001) (Fig. 3B). The decrease in amygdala Sox17 expression did not alter locomotion or anxiety-related behavior (Fig. 3C). The findings suggest that voluntary alcohol drinking triggers Sox17-related molecular cascades that may include OPC expansion and differentiation in the amygdala and consequently lead to an increase in alcohol intake.

### GWAS catalog and DisGeNET

To determine if our DEGs (FDR *p* < 0.05) are associated with AUD, we used an online GWAS catalog database (www.ebi.ac.uk/gwas). We found that 5 of our DEGs, including ATPase plasma membrane calcium transporting 1 (*Atp2b1*), heat shock protein family A member 4 (*Hspa4*), strawberry notch homolog 1 (*Sbno1*), solute carrier family member 7 (*Slc4a7*), and UBX domain protein 2b (*Ubxn2b*), from the Chronic Drinking group have previously been identified in GWAS of AUD. To further support our DEGs with previously reported findings in AUD, we next took advantage of the publicly available database, DisGeNET. Here we found that nuclear factor kappa B subunit 1 (*Nfkb1*) and neurotensin (*Nts*) from Acute Drinking group have been previously linked to AUD. Similarly, besides the 5 DEGs identified from the GWAS catalog, we found 5 more DEGs from our data set, including calcium/calmodulin-dependent protein kinase IV (*Camk4*), energy homeostasis associated (*Enho*), *Hdac2*, LDL receptor-related protein 6 (*Lrp6*), *Slc4a7*, and SLIT and NTRK like family member 2 (*Slitrk2*), which were previously linked to AUD in the literature.

## Discussion

The amygdala is highly sensitive to both chronic and acute alcohol drinking and alcohol-induced neuronal cellular plasticity within the amygdala circuits is well established. Since changes in neuronal physiology and neurotransmission may result from gene expression alterations, we here have characterized the transcriptome level responses to acute and chronic intermittent ethanol drinking. We subjected female B6 mice to a behaviorally well-validated 2-bottle choice drinking procedure and collected RNA samples from the amygdala. Using bioinformatics tools, we identified sets of significant DEGs, distinct GO and KEGG pathways, hub genes, and upstream transcriptional factors that are sensitive to ethanol drinking. Our results demonstrate that both acute and chronic ethanol drinking can impact similar biological processes including translational machinery, epigenetic modifications, synaptic plasticity, and neurological disorders. In addition, the findings also provide evidence indicating that alcohol drinking impacts on molecular and cellular alterations in non-neuronal cell types, such as OPC and oligodendrocytes in the amygdala [46].

Previous behavioral studies in both humans and animals suggested that alcohol tolerance and dependence can develop over several days or weeks, which seems to be a duration required to induce gene expression changes in response to alcohol exposure. Although there is a significant difference in alcohol intake between Acute and Chronic Drinking groups in our study, we did not observe FDR-significant differences in DEGs and GO/KEGG pathways between the Drinking groups. Rather a single bout of voluntary ethanol consumption resulted in molecular changes in the amygdala similar to those altered by repeated alcohol drinking. These results suggest that alcohol can trigger strikingly overlapping amygdala-specific gene expression changes regardless of the number of drinking episodes. Furthermore, acute alcohol drinking is sufficient to trigger critical molecular adaptations, possibly leading to future behavioral changes with repeated alcohol exposure. Notably, it has been reported that acute behavioral responses to alcohol have predictive value regarding risk for long-term alcohol drinking behavior in humans [47] and animal models [48].

Many of the genes we identified were of great interest given prior findings. Particularly, neurotensin (Nts) and its receptors have been implicated as contributing to the behavioral effects of alcohol in animal models. Chronic ethanol exposure increased Nts expression in the dorsal striatum [49], whereas ethanol decreased the expression of Nts receptors in both the nucleus accumbens (NAcc) and midbrain [50]. Furthermore, recent work has demonstrated that Nts-expressing neurons in the CeA contribute to the voluntary consumption of alcohol [40]. These findings are consistent with our results indicating an increase in Nts expression in Acute Drinking group, as the micropunches included the CeA in our samples. Interestingly, our recent studies demonstrated that the Nts receptor 2 (Ntsr2) is highly expressed in the BLA Thy1+ neurons that strongly project to the NAcc [51]. Therefore, our result provides an avenue of exploration at a circuit level into how the amygdala subnuclei, CeA-Nts and BLA-Ntsr2, may interactively mediate voluntary alcohol drinking behaviors.

Our study revealed many other well-known pathways affected by alcohol drinking. Amongst these, first, translational machinery including rRNA binding, ribosome, and ribosomal subunits seems to be positively affected by alcohol drinking. Consistent with previous studies in different brain areas [52], these results suggest that alcohol exposure also similarly affects translation of proteins in the amygdala, and leads to neuroadaptation via re-organization of synaptic structures, synaptic proteins and neurotransmitter receptors, such glutamate and GABA receptors [52]. Second, we found several enrichment terms related to chromatin remodeling, histone modification and DNA methylation in GO/KEGG analyses. Similarly, we also identified hub genes, mostly involved in histone deacetylation activity, including *Hdac2*. It was recently shown that there is increased *Hdac2* level and activity in the amygdala of *P* rats, an alcohol-preferring rat line, and acute systemic injection of ethanol decreased HDAC2 activity and subsequently reduced voluntary ethanol intake [53]. These results suggest that alcohol drinking affects gene expression by potentially regulating epigenetic alterations, particularly histone modifications via HDAC2. Third, we found that pathways related to neurodegenerative diseases, including Huntington disease and Parkinson disease, are enriched in the KEGG analysis. Since the brain is a major target for the actions of alcohol and heavy alcohol consumption has long been associated with brain damage as a risk factor [54], our study also confirms that alcohol consumption triggers similar molecular pathological pathways involved in neurodegenerative diseases. Fourth, one of interesting GO terms in our analysis is “myelin sheath”, indicating the pathway involved in myeline sheath formation and maintenance is affected by alcohol drinking. Our results support hypothesis that alcohol consumption leads to disruption in myelination-related gene expression, as previous studies reporting the detrimental effects of alcohol on white matter integrity and functions in animals and humans [55–60]. Similarly, genomic studies of alcohol also have found that functional enrichment of alcohol-sensitive myelination-related genes in rodents, primates, and human postmortem tissues [17, 21, 61–64]. However, our results contrast with those reports of the downregulation of myelination-related genes and proteins in cortical regions of chronic alcoholics. Instead, our analysis showed that gene expression in this pathway was increased in Acute and Chronic Drinking groups. A month-long drinking paradigm in the current study focusing on the amygdala may contribute to a difference in gene expression profile, reflecting a potential molecular recovery mechanism activated by myelination damages after alcohol drinking.

While co-expressed genes form functional networks, identifying upstream regulators of these genes and networks can provide insight into cellular function and lead to a potential therapeutic intervention. In our study, we observed that many of the DEGs from Acute and Chronic Drinking groups are regulated by *Sox17*, a transcription factor that regulates OPC proliferation and differentiation to oligodendrocytes via the Wnt/β-catenin signaling pathway [65, 66]. As oligodendrocytes provide myelin sheaths in the central nervous system, this finding is consistent with the results from our GO/KEGG analysis that identified “myelin sheath” as a key term. In addition, the downregulation of Sox17 in the amygdala prevented an increase in alcohol uptake over time, implicating that Sox17 mediates neural mechanisms underlying the escalation of alcohol drinking with the repeated alcohol access. To our knowledge, our study is the first to investigate the effects of alcohol drinking on an upstream regulator of OPCs and oligodendrocytes, which is an understudied form of cellular plasticity that may mediate behavioral outcomes such as alcohol drinking. Together these data, along with the above myelin sheath findings in our pathway analyses, provides strong evidence for a role of oligodendrocyte alterations in the aftermath of alcohol consumption.

In summary, we identified alcohol-sensitive, amygdala-associated candidate genes and pathways by genome-wide transcriptomic analyses. Consistent with previous gene expression studies, we found that voluntary alcohol consumption, regardless of the number of drinking episodes, results in similar gene expression changes in ribosome-related / translational pathways, myelination, chromatin-binding, and histone modification. These genes and pathways suggest convergence of human GWAS and molecular studies with amygdala transcription data from mouse drinking models. As the current study suggests alcohol-induced cell type-specific changes, future studies using advanced cell targeting techniques are warranted to validate the roles of identified genes in neural adaptation processes mediating the progression from acute to chronic alcohol intake.

## Supplementary information

Supplementary Materials
