# Supplementary material for: Genome-wide transcriptomics of the amygdala reveals similar oligodendrocyte-related responses to acute and chronic alcohol drinking in female mice
Source: Transl Psychiatry. 2022 Nov 12;12:476. doi: 10.1038/s41398-022-02231-2 (PMC9653459; doi:10.1038/s41398-022-02231-2)
Supplement: Supplementary file 1 — Supplementary Materials [file 41398_2022_2231_MOESM1_ESM.pdf]

SUPPLEMENTARY INFORMATION

Supplementary Methods.

Data Processing

The quality of raw sequencing reads was first assessed with FastQC (version 0.11.8, Babraham Institute, Cambridge, UK). Salmon (version 0.14.1) [67] was used to first build a transcriptome index using the combined reference transcriptome and non-coding RNAs for *Mus musculus* (GRCm38/mm10), and then to quantify the RNA-seq reads at the transcript level. The tximport (Bioconductor version 3.12) [68] was used to import the transcript-level abundances generated by Salmon, the estimated counts and the corresponding transcript lengths, and to summarize it into transcript-level and gene-level expression matrices. Gene-level expression matrices were generated using the GENCODE mouse annotation (www.gencodegenes.org) as reference (release M21). We next used ComBat-seq (version 3.38) [33] to adjust the batch effect by keeping the negative binomial distribution of RNA-seq reads count and the integer nature of the data. A pre-filtering step was then applied to the corrected gene counts where genes with less than ten counts on average across all the samples were filtered out, after which 16,260 genes were retained for the subsequent analysis. Fastq files have been submitted to the GEO database (accession number: GSE208014) for public access following publication of this manuscript.

Estimation of cell type abundances

Cell type abundances were estimated with BRETIGEA (version 1.0.3) [35] for each of the following cell types – neuron, astrocytes, microglia, oligodendrocyte, oligodendrocyte precursor cell (OPC), and endothelial cell. BRETIGEA contains thoroughly validated datasets containing brain cell type-specific marker genes. Using the '*brainCells'* function, with the filtered dataset

containing 16,260 genes as input, a sample-by-cell type matrix of estimated cell type proportion variables was generated. In addition to BRETIGEA, another computational cell type deconvolution tool, CIBERSORTx [69] was also used after building a signature matrix file for different cell types based on mouse single cell RNA sequencing data [36]. On the web interface, 500 permutations were chosen, and the quantile normalization option was disabled. Differences in cell type proportions between drinking groups were assessed with two-sample *t*-tests.

<u>Differential expression</u>

DESeq2 (version 1.30.1) [70] was used for downstream differential expression analysis using default parameters. DESeq2 performed an independent filtering step using the means of normalized counts as a filter statistic. A threshold for the filter statistic was found which optimized the number of P-values below a user-specified significance level. Using a nominal p-value cutoff of 0.05, 1300 and 1384 for Acute and Chronic Drinking groups, respectively, were retained as tentative DEGs for subsequent analyses. Principal component analysis (PCA) was constructed to calculate the coefficient of variation between groups directly using '*plotPCA*', a functionality in the package. Next, to trim potential false positive results, the more stringent false discovery rat (FDR)-adjusted p-value cutoff of 0.05 was used and 29 (Acute Drinking) and 97 (Chronic Drinking) DEGs were identified.

<u>Functional and Pathway Enrichment Analysis of DEGs</u>

The lists of tentative DEGs, with a nominal p-value cutoff of 0.05, were first divided into two sets, up- and down-regulated, and then function and pathway enrichment analysis were performed using clusterProfiler (version 3.18.1) [71]. Gene ontology (GO) analysis provided gene

annotations in biological processes, molecular functions, and cellular components. In addition, Kyoto Encyclopedia of Genes and Genomes (KEGG) analysis was carried out to gain more information on biological pathways related to diseases and drug targets.

GWAS Catalog and DisGeNET comparison

To acquire a better understanding of whether the significant genes obtained in this study were already implicated in previously published alcohol addiction studies, the list of significant genes from the DESeq2 analysis was compared with the list of significant genes in GWAS Catalog (release data: Oct 2019; www.ebi.ac.uk/gwas) and DisGeNET (release data: May 2020; www.disgenet.org) databases. The "*All associations v1.0.2 - with added ontology annotations, GWAS Catalog study accession numbers and genotyping technology*" dataset from the GWAS Catalog website and the "*ALL gene-disease associations*" dataset from the DisGeNET website were first downloaded and processed in R. From the GWAS Catalog dataset, the columns of interest "*DISEASE/TRAIT*" and "*REPORTED GENE*" were retained, while from the DisGeNET dataset, "*geneSymbol*" and "*diseaseName*" were retained. The analysis was performed with disease terms containing "*alcohol*" from both "*DISEASE/TRAIT*" and "*diseaseName*" columns, after which, the disease terms containing "*nonalcohol/non-alcohol*" were filtered out. The significant genes (FDR-adjusted p value < 0.05) from the DESeq2 analysis were then matched with the resulting genes from both the GWAS Catalog and DisGeNET datasets, to get the overlapping/common genes.

Identification of Hub genes and Regulatory Transcription Factors

To visualize protein-protein interactions networks among DEGs, the Search Tool for the Retrieval of Interacting Genes (STRING) online database (v11.0) [45] was used with DEGs with

the criteria of adjusted p-value cutoff < 0.05. The same sets of DEGs were also used with GeneGo MetaCore (Clarivate Analytics, PA) to detect upstream transcription factors. The Transcriptional Regulatory Relationships Unraveled by Sentence-based Text mining (TRRUST) (v2) [72] online database was also utilized to discover transcriptional regulatory networks.

<u>Supplementary Figure Legends.</u>

<u>Supplementary Figure 1.</u> Fluid consumption levels and the number of DEGs between Drinking groups. (**A**) Ethanol intake over 24 hours on water/20% EtOH drinking days separated by cohort. (**B**) Water intake over 24 hours on water/water drinking days.

<u>Supplementary Figure 2.</u> Assessments of gene expression profiles between batches. (**A**) PCA plot showing separation on batch over the first two principal components, explaining 89% of the variation in total. There was no outlier sample within each batch. (**B**) The top 100 genes were selected based on adjusted P-valeuds from batch 1, and fold changes for these genes were compared in between two batches. r: Pearson's r. (**C**) Same as panel (B) but using genes selected based on adjusted P-values from batch 2. (**D**) After processing raw counts using ComBat-seq, the first two principal components from two batch was plotted. The PCA plot explains 52% of the variation in total, revealing no segration among Drinking groups.

<u>Supplementary Figure 3.</u> Cell type abundances and Venn-diagrams of DEGs among Drinking groups. (**A**) Bar graphs showing the estimated cell type abundances for 7 relevant cell types as determined by cell type deconvolution analysis, CIBERSORT. (**B**) Overlap Venn Diagrams of differentially expressed genes (DEGs) for comparisons across all Drinking groups.

<u>Supplementary Figure 4.</u> GO and KEGG pathway enrichment analysis of DEGs.
The ordinate represents the GO or KEGG terms, the upper abscissa indicates the number of genes in the GO/KEGG terms, and the lower abscissa indicates the level of significance of the enrichment

(gray bar, FDR = 0.01). (**A**) Genes were categorized with the Molecular Function domain. (**d**) The top 5 enriched pathways in the KEGG pathway analysis.

<u>Supplementary Figure 5.</u> The protein-protein interaction (PPI) network analysis using STRING database

The 97 DEGs from the Chronic Drinking group were input into STRING database and archived 95 nodes and 38 edges, with PPI enrichment p-value < 0.01. Magenta and turquoise lines indicate known protein-protein interactions; black and greenish yellow lines indicate interactions based on co-expression data and textmining, respectively.

Supplementary Figure 1.

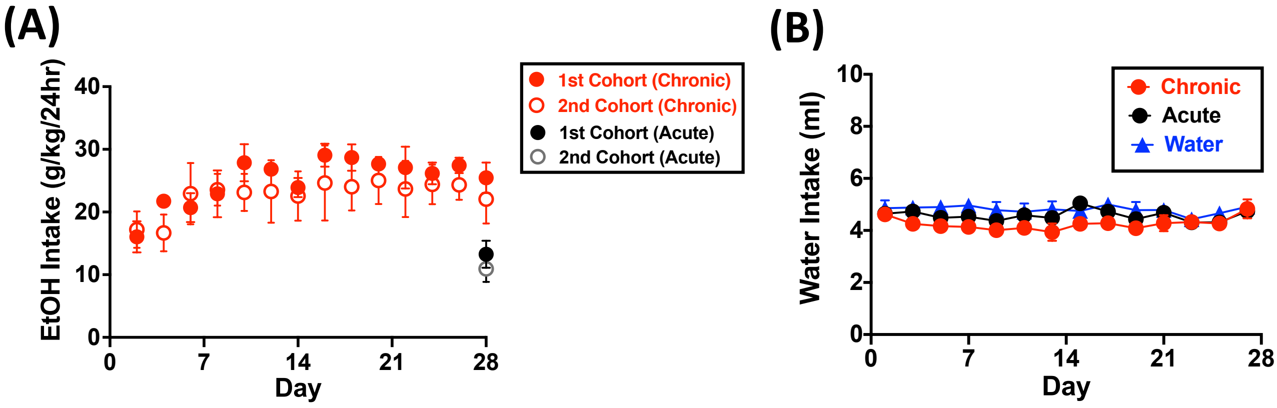

Supplementary Figure 2.

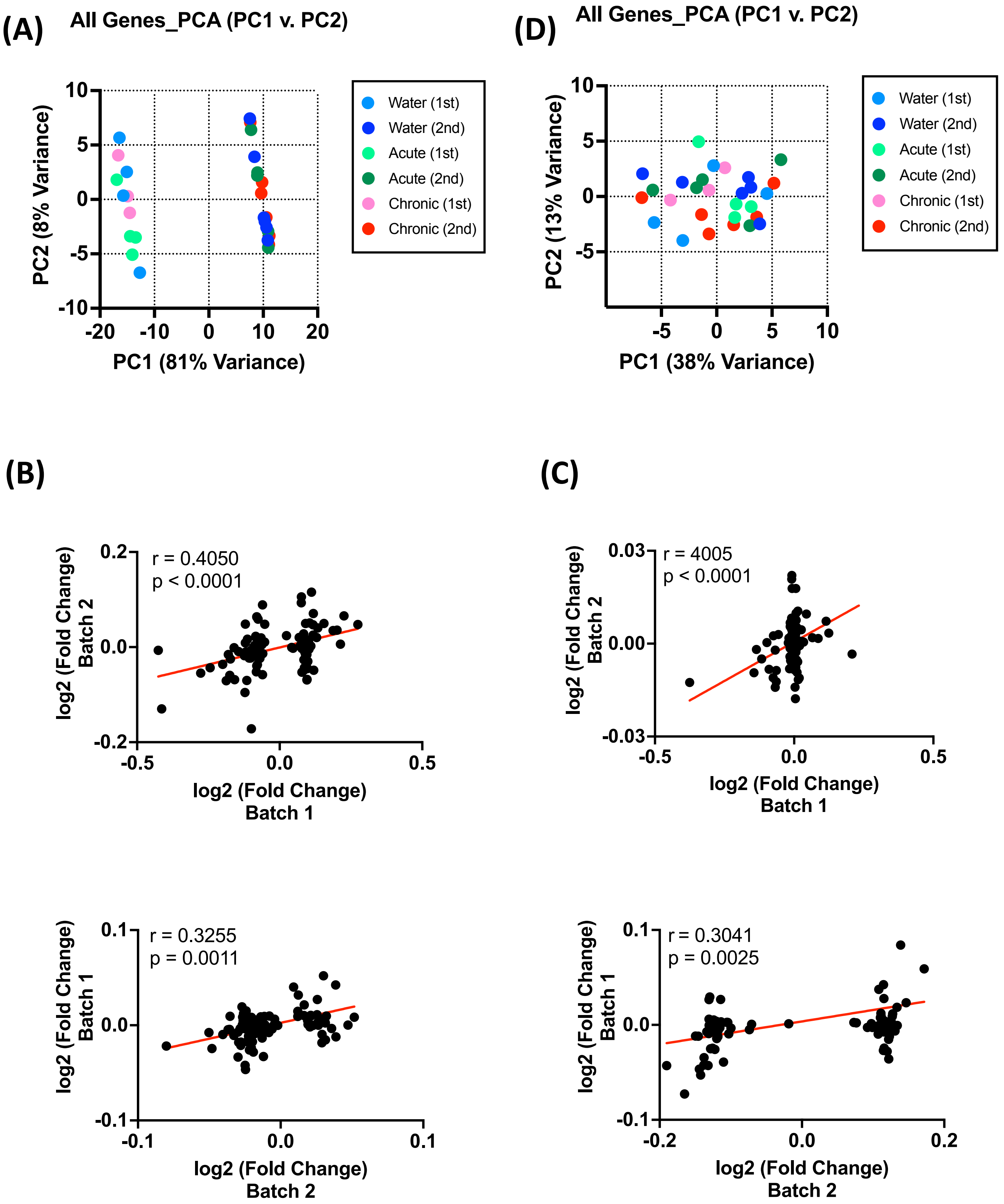

Supplementary Figure 3.

(A)

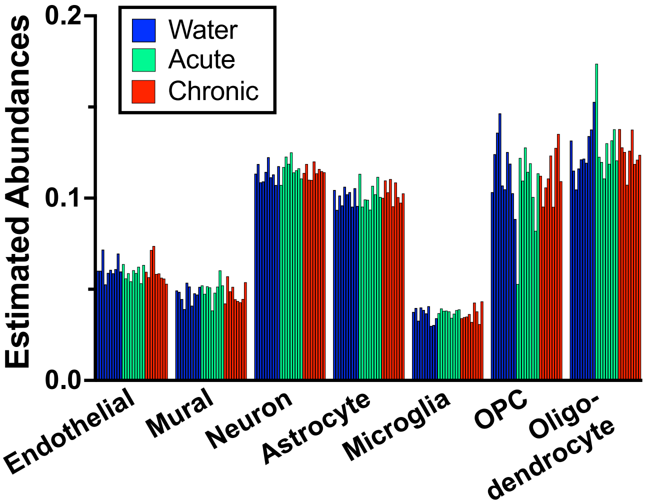

(B)

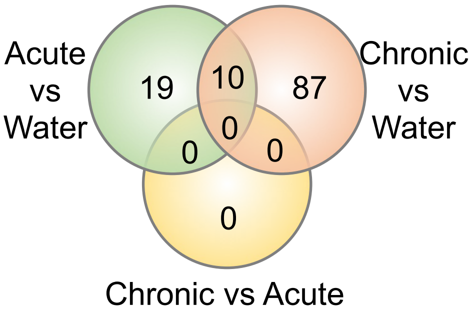

Supplementary Figure 4.

(A)

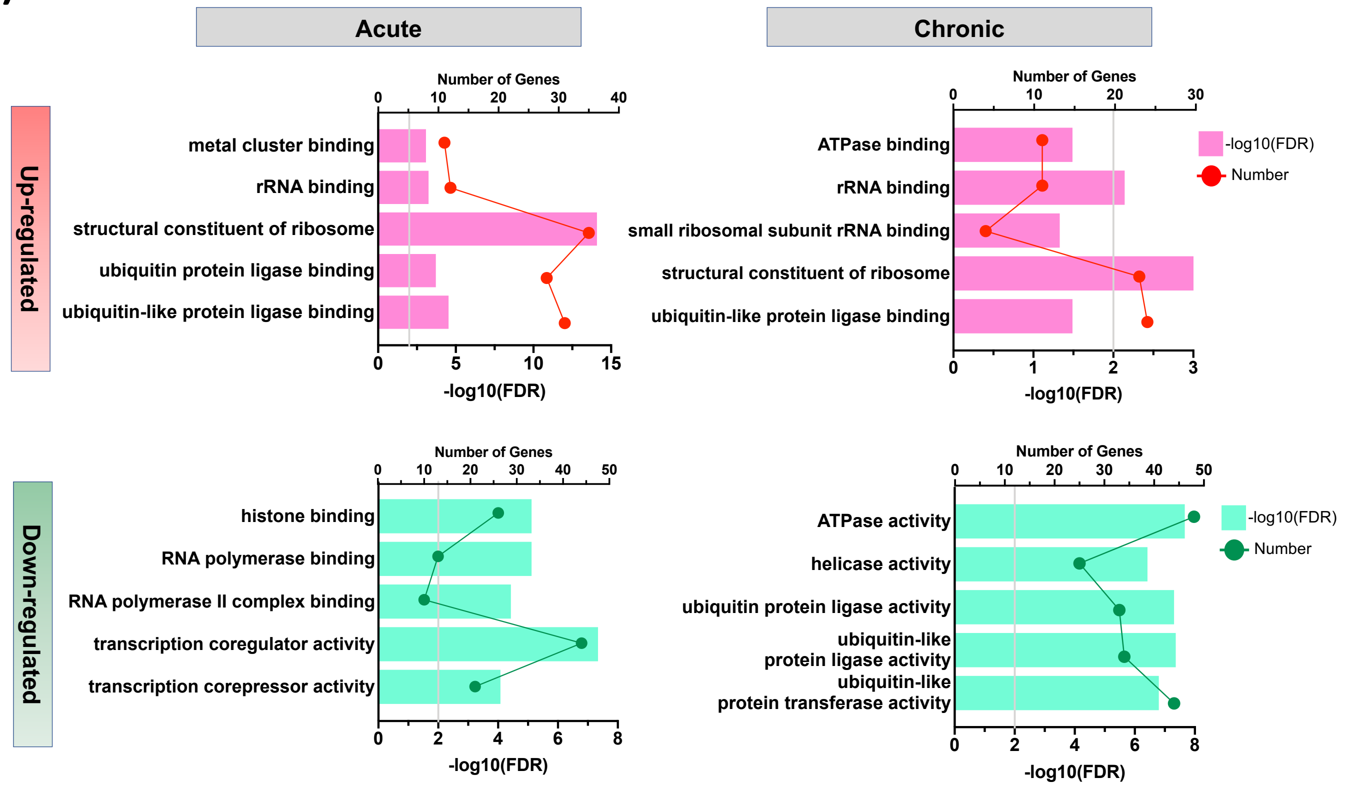

(B)

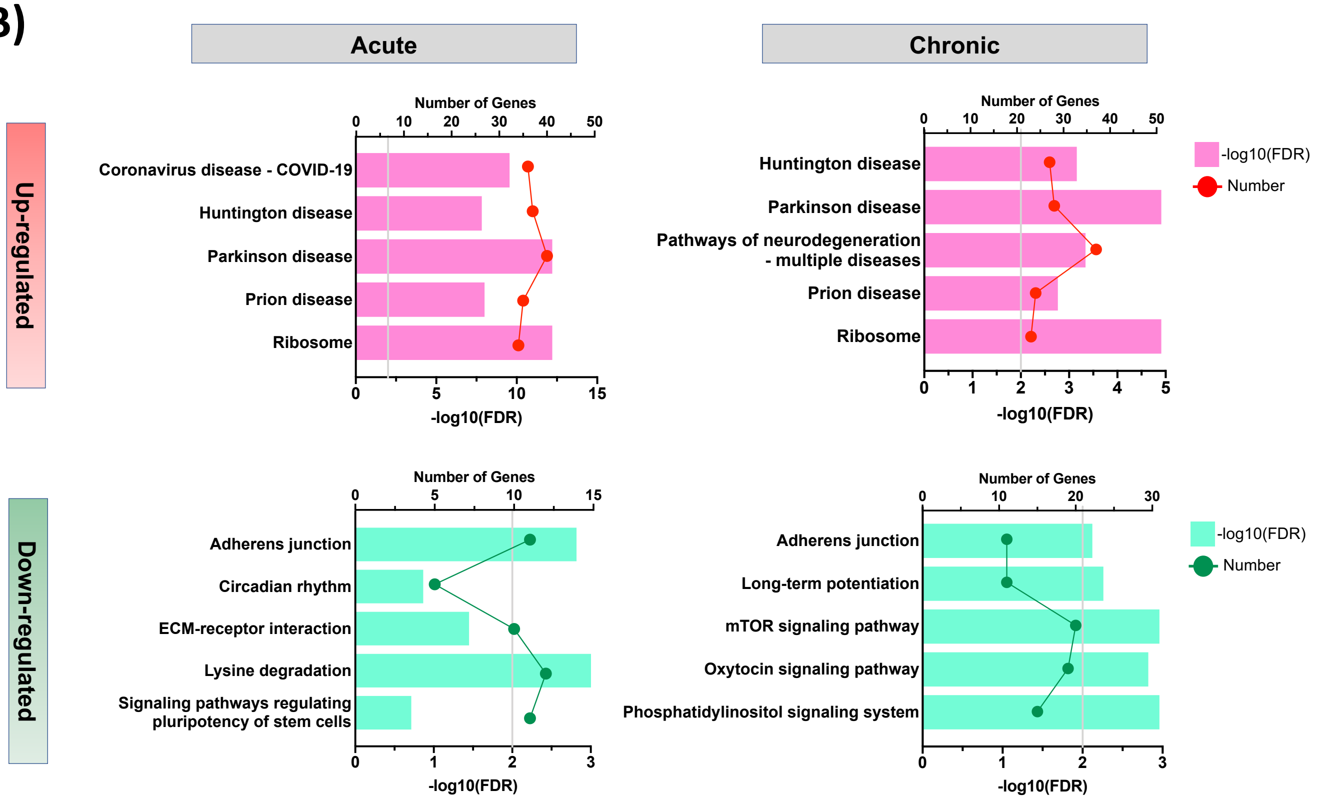

Supplementary Figure 5.

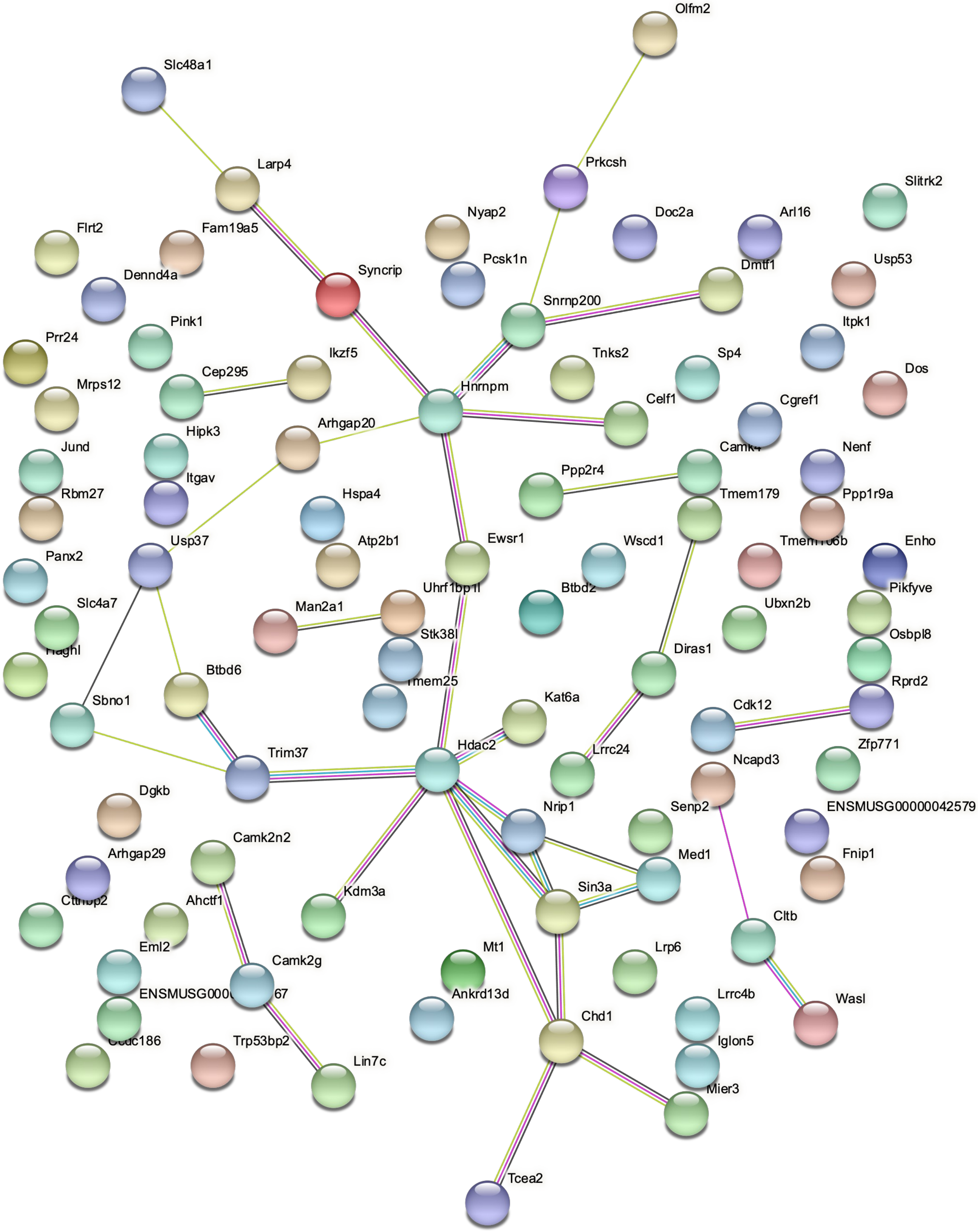

Supplementary Table 1. Primers for genes of interest and housekeeping gene, *Actb* for qPCR

Gene	5' Primer	3' Primer
Bcl	GGTCCTCAGCTCTGGAAAAA	AGGTTGTGTGTGCCAGTTACC
Btg2	GCGAGCAGAGACTCAAGGTT	CCTTTGGATGGTTTTTCTGG
Haghl	GGACTCACCAGCCCTCTTCT	TTGGCCAAGCTCTGGTACAT
Kdm3a	CATTGGAGCAAAACTTCCTCA	TGGTTTTGTTCTCGGTACTTCA
Lrrc24	GCTGGATTTCACCTTCTTGC	GCCTGGTCCTCCAGTAATTC
Nenf	GGATCCAGCAGACCTCACTC	TGGCTTTGTACACCTTGCTG
Nts	TCCAGCTCCAGAAAATCTGC	CCTTCTCGTTTTTATCATTGACG
Hdac2	ACCACATGCACCTGGTGTTCA	TCGCAAGCTATCCGTTTGTCTG
Hnrnpm	GCTGGAAGACTTGGAAGCAC	CCCTTTGGTAAAGCCCTCTC
Sin3a	AACACCTGGCCAAGTTCATC	TGCTGACTGGCTGGAGTATG
Chd1	AGGTTCCAAGGGCAGTACCT	CACTCGGTTGCCTCTTTCTC
Actb	CCAACCGTGAAAAGATGACC	ACCAGAGGCATACAGGGACA

Supplementary Table 2. Validation of DEGs by qPCR

		**RNA-seq**		**qPCR**		
	**Gene**	**Fold change**		**Fold change**		
		Acute	Chronic	Water	Acute	Chronic
**Up-regulated**	*Bc1*	1.000	1.167	1.00 ± 0.11	1.05 ± 0.18	1.27 ± 0.13
	Btg2	1.346	1.154	1.00 ± 0.09	1.40 ± 0.15*	1.45 ± 0.12*
	Haghl	1.131	1.153	1.00 ± 0.10	1.35 ± 0.10*	1.57 ± 0.14**
	Lrrc24	1.139	1.159	1.00 ± 0.13	1.37 ± 0.10*	1.04 ± 0.12
	Nenf	1.149	1.182	1.00 ± 0.09	1.12 ± 0.28	1.34 ± 0.10*
	Nts	1.462	1.180	1.00 ± 0.08	1.52 ± 0.18*	1.25 ± 0.08
**Down-regulated**	*Kdm3a*	0.891	0.850	1.00 ± 0.09	0.67 ± 0.07*	0.71 ± 0.05
	Hdac2	0.996	0.932	1.00 ± 0.07	0.99 ± 0.12	0.94 ± 0.11
	Hnrnpm	0.918	0.887	1.00 ± 0.10	0.95 ± 0.07	0.92 ± 0.05
	Sin3a	0.954	0.922	1.00 ± 0.06	0.97 ± 0.12	0.95 ± 0.10
	Chd1	0.951	0.904	1.00 ± 0.12	0.98 ± 0.17	0.97 ± 0.06

Supplementary Table 3. Summary of RNA expression data sets used for cell type-specific expression and number of DEGs

GEO #	Species	Tissue	Data Type	Drinking Condition	Cell Type					
					Neuron	Astrocyte	Microglia	OPC	Oligodendrocyte	Endothelial Cell
GSE9566	Mouse	Forebrain	Bulk RNA	Acute	NTS, NELL1					
				Chronic		STK38L				
GSE52564	Mouse	Cerebral Cortex	Bulk RNA	Acute						
				Chronic				TMEM179	NOL3, STRN	
GSE103976	Mouse	Medial Amygdala	Single Cell	Acute	PCSK1N, ATP1B1					KLF2
				Chronic	PCSK1N, GAP43, ATP1B1	SLC6A1			TUBB4A	
GSE144136	Human	Prefrontal Cortex	Single Cell	Acute		SLC39A11, CAMK2G			SLC39A11, TJP1	
				Chronic		CAMK2G, OLFM2, ENHO, CNTFR, ITGAV, PREX2, SORBS1, CEPO85L	USP53, SLC4A7, PICALM	WSCD1, AMZ1, CTTNBP2, PPP1R9A, ITGAV, MMP16, PREX2	NENF, SLC48A1, TUBB4A, HDAC11, APLP1, MAN2A1, LRP6, MMP16, STRN, DSEL, PICALM	

A list of upregulated (red) and downregulated (green) DEGs appeared in cell type-specific RNA expression data sets from previous studies [36, 41-43].
